# Complement factor H: a novel innate immune checkpoint in cancer immunotherapy

**DOI:** 10.3389/fcell.2024.1302490

**Published:** 2024-02-08

**Authors:** Ruchi Saxena, Elizabeth B. Gottlin, Michael J. Campa, Ryan T. Bushey, Jian Guo, Edward F. Patz, You-Wen He

**Affiliations:** ^1^ Department of Integrative Immunobiology, Duke University School of Medicine, Durham, NC, United States; ^2^ Department of Radiology, Duke University School of Medicine, Durham, NC, United States; ^3^ Department of Pharmacology and Cancer Biology, Duke University School of Medicine, Durham, NC, United States

**Keywords:** complement, complement factor H, immunosuppression, tumor micro-environment, immune checkpoint, immunotherapy

## Abstract

The elimination of cancer cells critically depends on the immune system. However, cancers have evolved a variety of defense mechanisms to evade immune monitoring, leading to tumor progression. Complement factor H (CFH), predominately known for its function in inhibiting the alternative pathway of the complement system, has recently been identified as an important innate immunological checkpoint in cancer. CFH-mediated immunosuppression enhances tumor cells’ ability to avoid immune recognition and produce an immunosuppressive tumor microenvironment. This review explores the molecular underpinnings, interactions with immune cells, clinical consequences, and therapeutic possibilities of CFH as an innate immune checkpoint in cancer control. The difficulties and opportunities of using CFH as a target in cancer immunotherapy are also explored.

## 1 Introduction

The immune system is crucial in identifying and eradicating cancer cells (Fridman, 2012; Fridman et al., 2017). Tumors, however, have developed a variety of ways to elude immune surveillance, create an immunosuppressive microenvironment, and often coopt the immune system to promote tumor growth (Vinay et al., 2015; Abbott and Ustoyev, 2019). The concept of immunological checkpoints, which has emerged as a ground-breaking field, has highlighted the relevance of immune mechanisms that inhibit the host response (He and Xu, 2020; Pisibon et al., 2021; Guo et al., 2023). While immune checkpoints like PD-1/PD-L1, and CTLA4 have attracted a lot of attention, more recent data suggests that fundamental innate immune mechanisms including the complement system are also involved in immune escape mechanism in cancer (Parente et al., 2017; Moore et al., 2021).

The complement system constitutes an essential component of the innate immunity comprising of over 32 different proteins, including membrane proteins, serum proteins, and serosal proteins. Three distinct mechanisms can activate the complement system: the alternative pathway (AP), the classical pathway (CP), and the lectin pathway (LP) (Ricklin and Pouw, 2021; Kemper et al., 2023). While the CP and LP are initiated by antibody- and carbohydrate-mediated recognition processes, respectively, the AP is activated by hydrolysis of C3 and is constitutively active. Though the three pathways differ in the initiation of the complement cascade, they converge at the formation of C3 convertase (C4bC2b in CP and LP and C3bBb in AP) resulting in cleavage of C3 to C3a and C3b. Furthermore, by binding C3b, C3 convertases forms C5 convertases (i.e., C4bC2aC3b in CP and LP and C3bBbC3b in the AP), resulting in the assembly of cell lytic, membrane-attack complex (MAC, C5b-9). Membrane bound C3b also act as opsonin and triggers ingestion by phagocytosis. Furthermore, the anaphylatoxins C3a and C5a, formed as split products of C3 and C5 cleavage, associate with the cells of the innate and adaptive immune system to induce chemotactic and inflammatory responses (Ling and Murali, 2019; Kemper et al., 2023).

Complement factor H (CFH) is the most important inhibitory regulator of AP activation. It is a serum protein that plays a crucial function in suppressing complement activation on cells and in the extracellular matrix of host tissues (Ferreira et al., 2010) by binding to cell surfaces via glycosaminoglycans (Blaum et al., 2015; Langford-Smith et al., 2015). While liver is the predominant source of CFH (Schwaeble et al., 1987), it is also produced by other cell types (Brooimans et al., 1990; Chen et al., 2007; Licht et al., 2009; Ferreira et al., 2010; Sakaue et al., 2010; Tu et al., 2010). In addition, a truncated form of CFH, known as factor H-like protein 1 (FHL-1), produced by alternative splicing of the *cfh* gene (Ripoche et al., 1988), can be found locally, such as in retinal pigment epithelial cells and the liver (Clark et al., 2014). CFH competes with factor B for binding to C3b and has three complement-regulating functions: 1) prevents the formation of C3 convertase (C3bBb) via the AP; 2) accelerates the dissociation of already formed C3bBb; 3) serves as a cofactor for the serine protease complement factor I (CFI) making C3b susceptible to cleavage leading to the formation of iC3b (Makou et al., 2013). When bound to cell surface, the iC3b fragment undergoes further hydrolysis by CFI and membrane-bound cofactors like CR1, resulting in the release of C3dg and C3d. While C3b deposition initiates series of reactions culminating in the formation of MAC, its proteolytic degradation products, iC3b, C3dg, and C3d lead to lymphocyte activation and maturation by interacting with their receptor, complement receptor 2 (CR2) on immune cells like B cells and DCs (Kalli et al., 1991; Nagar et al., 1998; Carroll, 2004; Lyubchenko et al., 2005; Carroll and Isenman, 2012; Merle et al., 2015). Thus, by preventing the production and amplification of C3 convertases and encouraging the decay of already produced convertases, CFH plays a crucial part in controlling amplification of the complement system (Jozsi et al., 2019). By interacting with a different complement protein, including C3b and C3d, as well as cell surface glycosaminoglycans (GAGs) and complement receptor 3 (CR3), CFH prevents complement activation. However, dysregulation of CFH expression and function in the context of cancer has also been linked to immune evasion, fostering tumor development and metastasis (Jozsi and Uzonyi, 2021).

## 2 Dysregulation of CFH in cancer and impact on clinical outcomes

CFH is overexpressed on many different types of cancer cells and is usually associated with poor prognosis. The first report of the association of CFH with cancer was published in 1998 where it was demonstrated that the presence of CFH on lung cancer cells makes them resistant to complement mediated lysis (Varsano et al., 1998). Since then, several reports have confirmed the role of CFH in various solid tumors including glioblastoma (Junnikkala et al., 2000), bone cancer (Fedarko et al., 2000), ovarian cancer (Junnikkala et al., 2002), colon cancer (Wilczek et al., 2008), cutaneous squamous cell carcinoma (Riihila et al., 2014) and breast cancer (Smolag et al., 2020). In contrast, a few reports have shown the anti-tumoral effect of CFH. Bonavita et al. demonstrate that in a mouse model of sarcoma, CFH exerts antitumoral effect by inhibiting the production of anaphylatoxins thus creating an immunosuppressive environment (Bonavita et al., 2015). Another study reports spontaneous hepatic tumor formation in aged mice with CFH deficiency (Laskowski et al., 2020). This was presumably due to life-long inflammation or potentially due to non-canonical effects of CHF; it is not clear if this finding would be relevant to humans undergoing treatment with an anti-CFH antibody. Thus, CFH has become a therapeutic target of interest, although the exact way to target CFH without off-target effects has been challenging.

CFH is also produced by cells other than the liver, including cancer cells. In renal cell carcinoma, intracellular CFH has been reported to drive tumor growth independent of the complement cascade (Daugan et al., 2021). It has been shown that CFH is upregulated in lung cancer, and that this overexpression is associated with larger tumors, lymph node metastases, and worse overall survival (Ajona et al., 2004; Cui et al., 2011). Increased CFH expression is also linked to larger tumor size, metastasis, and late stage tumors in breast cancer (Smolag et al., 2020). Further, CFH levels are elevated in cutaneous squamous cell carcinoma and overexpression is linked to immunosuppression (Johnson et al., 2022). Increased CFH expression has also been detected in tumor tissues relative to neighboring normal tissues in this disease. CFH dysregulation in cancer has significant implications for clinical outcomes. Increased CFH level in the tumors of lung adenocarcinoma patients is linked to poorer overall and disease-free survival and serves as a prognostic marker (Cui et al., 2011). Additionally, CFH may function as a prognostic biomarker in other malignancies including cutaneous squamous cell carcinoma (Johnson et al., 2022). Furthermore, it has been linked to the invasion and spread of cancer cells. Small extracellular vesicles, called exosomes, secreted by cancer cells can promote metastasis by delivering protein and mRNA cargo to other cancer cells and to non-cancer cells at distant sites, helping to prepare “premetastatic niches” where cancer cells can seed. Mao et al. have shown that CFH on tumor extracellular vesicles stimulates tumorigenesis and metastasis (Mao et al., 2020). Another study by Bushey et al. confirms that CFH-containing exosomes, secreted from tumors that express CFH, may be shielded from complement-dependent destruction by their CFH and a higher level of these exosomes has been linked to higher metastatic potential of cancer cell lines (Bushey et al., 2021).

Interestingly, early-stage non-small cell lung cancer patients who do not develop metastasis or recurrence after surgical resection had autoantibodies against CFH. These anti-CFH autoantibodies were found to recognize a conformationally unique CFH epitope hypothesized to be presented on the surface of tumor cells (Amornsiripanitch et al., 2010; Campa et al., 2015). The autoantibodies inhibited CFH binding to lung cancer cells, increased C3b deposition, and induced complement mediated lysis of tumor cells (Campa et al., 2015) and also served as prognostic marker for stage I NSCLC (Gottlin et al., 2022).

## 3 CFH functions as innate immune checkpoint

CFH is a key component in the process of cancer immune evasion (Ricklin et al., 2010). Multiple studies have established that cancer cells use CFH to undermine complement-mediated immune responses and enhance tumor growth and progression (Afshar-Kharghan, 2017; Revel et al., 2020). The importance of CFH in age-related macular degeneration and its connection to cancer are highlighted by Riihilä et al. (Riihila et al., 2014) who discuss how CFH overexpression may help cancer cells evade the immune system. CFH has been shown to create immunosuppressive effects by preventing complement activation, impairing antigen presentation, and encouraging regulatory T cell development (Parente et al., 2017). It also impacts immune cell trafficking, cytokine production, and tumor-associated macrophage polarization, contributing to an immunosuppressive tumor microenvironment (TME) (Wang et al., 2016; Parente et al., 2017; Bushey et al., 2023). The following five mechanisms have been proposed to explain how CFH helps cancer cells to evade complement-mediated toxicity or immune responses and advance tumor growth.

### 3.1 CFH causes complement evasion

As discussed previously, the activation of the complement pathway through CP, LP and AP results in the production of complement components that participate in various effector functions. These include opsonization, inflammation, and direct lysis of target cells. In the context of cancer, the complement system’s role extends beyond its traditional function, as it has been implicated in cancer immune evasion and tumor progression (Ajona et al., 2019b; Senent et al., 2022). Studies have demonstrated the involvement of complement in cancer immune surveillance. For instance, Reis et al. provide an extensive review on the role of complement in cancer immunotherapy, highlighting its importance in tumor recognition, inflammation, and clearance (Reis et al., 2018). Zhang et al. discuss the implications of the complement system in tumor development, prevention, and therapy, emphasizing the role in immunosurveillance and the potential for targeting complement components in cancer treatment (Zhang et al., 2019). These studies collectively underscore the critical role of the complement system in cancer immune surveillance and its potential as a target for cancer immunotherapy.

CFH gives cancer cells the ability to sabotage complement-mediated immune responses and advance tumor growth as discussed above. The formation of the membrane attack complex (MAC) and consequent cell lysis are both prevented by CFH’s ability to attach to the surfaces of cancer cells and suppress complement activation (Parente et al., 2017). Additionally, CFH can obstruct opsonization, which is necessary for phagocytic cells to recognize and destroy cancer cells. CFH binds to C3b and thus prevents C3b from adhering to cancer cells and inhibiting phagocytosis. Further, by modulating the complement cascade, CFH can limit the production of pro-inflammatory complement fragments C3a and C5a and suppress immune cell-mediated tumor destruction. In addition, CFH prevents the cleavage of C3b and thus the formation of cleavage products displaying the C3d moiety that serves as a ligand for CR2 receptors on B cells and DCs. The binding of C3d to CR2 receptor on immune cells is important for activation and affinity maturation of lymphocytes (Alcorlo et al., 2015; De Groot et al., 2015). Due to these evasion tactics, cancer cells are better able to survive and proliferate by evading immune surveillance.

### 3.2 CFH impairs antigen presentation, T cell expansion, and B cell functions

CFH has been found to interfere with antigen presentation, a critical step at the beginning of adaptive immune responses against cancer cells. Dendritic cells (DCs) can bind CFH and lose their capacity to effectively deliver tumor antigens to T cells. This reduced antigen presentation restricts the activation and growth of tumor-specific T cells, suppressing the adaptive anti-tumor immune response and fostering immunological tolerance (Olivar et al., 2016; Dixon et al., 2017). CFH also enhances the differentiation of anti-inflammatory and tolerogenic monocyte derived DCs. These immature DCs show lower expression of maturation markers and costimulatory molecules, decreased production of pro-inflammatory Th1-cytokines like IL-6, IL8, IL-12, TNF, IFN-γ, and favored immunomodulatory cytokines production including TGF-β and IL-10. These DCs do not cause activation of allo-stimulated T cells and increased the production of regulatory T cells (Tregs) (Olivar et al., 2016).

In addition, CFH directly engages with T cells and inhibits their activation, proliferation, and effector activities (Wang et al., 2016). CFH inhibits intratumoral effector T cell function through direct binding to its receptor CR3 or indirectly through inhibiting production of complement activation products C3a and C5a. It has been shown that CR3 is expressed on activated CD4^+^ and CD8^+^ T cells (Gray and Horwitz, 1988; Savary and Lotzova, 1992; Hamann et al., 1997; Wagner et al., 2001) and a majority of CD8^+^ TILs from melanoma (Hersey and Jamal, 1990) and pancreatic cancer (Ademmer et al., 1998). The upregulated CR3 delivers a negative signal to effector T cells because not only is CR3 expression associated with dysfunctional (CD28^−^, CD4low, CD8low, or CD57^+^) T cells in different diseases (Dianzani et al., 1994; Mollet et al., 1998) but also direct engagement of CR3 on T cells by C3bi-coated zymosan was shown to inhibit T cell proliferation (Wagner et al., 2001). Recent reports strongly support the premise that tumor-associated/derived CFH binds to CR3 on CD4^+^ and CD8^+^ TILs and directly inhibits their expansion. Further, it is known that locally produced C3a/C5a signaling through Ca3R/Ca5R on T cells is required for CD28 upregulation and T cell activation (Lalli et al., 2008; Strainic et al., 2008). Since CFH inhibits the production of these anaphylatoxins (Ajona et al., 2004) further T cell activation is impeded. However, the role of C3a/C5a in T cell function tumor immunity is still controversial (Reis et al., 2018; Ajona et al., 2019a; Wang et al., 2019).

Merle et al. discovered that CFH also inhibits B cell activation and function. (Merle et al., 2015). The C3 split product, C3d when conjugated with antigen interacts with its receptor CR2 on B cells and follicular DC to induce both B cell activation and follicular DCs mediated B cell affinity maturation thus presenting these antigens in germinal centers to incite effector and memory B cell responses (Carroll and Isenman, 2012; Merle et al., 2015). CFH interferes with the production of complement split product C3d and thus inhibits B cell responses. Further, uninhibited systemic complement activation is induced by loss of CFH resulting in increased production of C3 fragments with concomitant elevated surface levels of CR2 on mature B cells associated with increased BCR signaling (Kiss et al., 2019).

### 3.3 CFH promotes regulatory T cell expansion

Though the direct interaction of CFH with Tregs has not been established, the proliferation and activation of Tregs in the TME have been linked to CFH overexpression. It has been shown that tumor cell-expressed indoleamine 2,3-dioxygenase 1 increased CFH and FHL-1 expression independent of tryptophan metabolism resulting in Treg proliferation and activation. Further, increased expression levels of CFH and FHL-1 levels were associated with poorer survival in glioblastoma patients (Zhai et al., 2021). One possible explanation for the effect of CFH on Tregs is that CFH inhibits production of C3d. C3d binds to CR2 on intratumoral Tregs (itTregs) and cause suppression of Id2 expression which in turn triggers apoptosis and loss in function of itTregs. Amplified CFH expression or activity creates an environment that is more immunosuppressive and favorable to Treg accumulation and function. Platt et al., have shown that C3d in the tumor augments anti-tumor immunity via eliminating itTregs by binding and signaling through its cognate receptor CR2 on itTregs (Platt et al., 2017). Furthermore, increased CFH expression is associated with increased prevalence of Tregs and an immunosuppressive TME in cutaneous squamous cell carcinoma (Johnson et al., 2022).

### 3.4 CFH modulates immune cell trafficking and function

CFH has the capacity to control cytokine production and immune cell trafficking in the TME. It can influence the recruitment and activation of other immune cells, such as neutrophils and macrophages (Ricklin and Pouw, 2021). CFH binds to CR3 receptors (CD11b/CD18) on neutrophils via its SCR7 and SCR19-20 domains (Avery and Gordon, 1993; DiScipio et al., 1998), to modulate cell activation and function (Losse et al., 2010). It is known that complement pathways and neutrophils serve to activate each other and the AP acts as a positive feedback amplification of neutrophil activation (Camous et al., 2011; Halbgebauer et al., 2018). Studies have shown that neutrophils with immunosuppressive properties expand in the TME and are associated with poor prognosis. The neutrophils in the TME secrete proteases, chemokines and cytokines attracting other tumor promoting immune cells or immunosuppressive T cells along with ROS generation and formation of neutrophil extracellular traps (NETs) facilitating cancer progression. These neutrophils can serve as biomarkers for progression and therapy response in cancer patients and may be used as targets to augment the efficacy of anti-cancer therapy (Masucci et al., 2019; Wu et al., 2020). Further, Zhao et al. have shown that a higher ratio of neutrophil-to-lymphocyte is associated with adverse overall outcome in many solid tumors (Wu et al., 2020; Zhao et al., 2020). Interestingly, there are reports to show that CFH not only facilitates neutrophil recruitment, but it also has an anti-inflammatory effect induced by the formation of neutrophil extracellular traps (NETs) (Schneider et al., 2016; Chen et al., 2018). In addition, CFH treatment of neutrophils induces reactive oxygen species (ROS) production and degranulation in neutrophils activated by PMA, fibronectin plus b-glucan, or anti-neutrophil cytoplasmic autoantibody, thus inhibiting neutrophil apoptosis (Kasahara et al., 1997).

Further, there is evidence to show that neutrophils and monocytes also engage CFH through membrane bound molecules such as integrins (e.g., αIIbβ3) and L-selectin (Lambris et al., 1980; Malhotra et al., 1999; Kang et al., 2012), that are known CFH receptors. In addition, CFH possibly binds to CR4 receptors on macrophages and DCs and exhibits similar cellular effects as binding to CR3 receptors on neutrophils (Parente et al., 2017). These additional interactions mediate the control of cell adhesion and migration, and cytokine production confirming that CFH has a direct anti-inflammatory and tolerogenic effect on intra-tumoral leukocytes.

CFH can also enhance Myeloid- derived suppressor cells (MDSC) accumulation, expansion, and immunosuppressive functions within the TME, contributing to immune evasion, probably via direct interaction with CR3 receptors (Sun et al., 2012; Parente et al., 2017) as discussed below. The role of MDSC accumulation in the TME in cancer progression has been confirmed by several studies (Pillay et al., 2013; Wu et al., 2020), and the importance of targeting these cells for cancer immunotherapy has also been demonstrated (Law et al., 2020; Li K. et al., 2021; Li X. et al., 2021). Dysregulation of CFH can lead to immunological escape from tumors by impairing immune cell homeostasis and changing the ratio of pro-inflammatory to anti-inflammatory cytokines (Jozsi et al., 2019; Smolag et al., 2020).

### 3.5 CFH promotes tumor-associated macrophage polarization

CFH can promote tumor-associated macrophages (TAMs) polarization towards an immunosuppressive M2-like phenotype, characterized by the release of immunosuppressive and anti-inflammatory cytokines. It favors differentiation of CD14^+^ monocyte into IL-10 producing (HLA-DRlowPD-L1hi) macrophages with immunosuppressive properties. CFH binds to unidentified receptor(s) on monocytes surface through it SCR19-20 region that leads to its internalization triggering downstream intracellular events to transform the transcriptome into an immunosuppressive signature. (Smolag et al., 2020). This polarization also favors tumor growth and creates an immunosuppressive TME.

In summary, CFH mediates immune evasion by cancer cells through a variety of cellular and molecular pathways. Because of its overexpression, inhibition of opsonization, and support for an immunosuppressive TME, CFH helps cancer cells resist complement-mediated and complement-independent immune responses. Understanding these pathways lays the groundwork for the creation of innovative therapeutic approaches that target CFH to boost antitumor immune responses and enhance cancer treatment results.

## 4 Targeting CFH as an immune checkpoint for cancer immunotherapy

Targeting CFH and its related molecular pathways is a novel therapeutic approach to modify the TME, improve anticancer immune responses, and decrease tumor growth and spread. Multiple strategies could be used for targeting CFH for anti-cancer therapies. Use of monoclonal antibodies (mAbs) designed to specifically target CFH could neutralize its immunosuppressive effects and inhibit cancer progression. These mAbs can potentially block CFH’s interactions with complement components, permit complement activation of tumor cells, while driving anti-tumor immune program. Campa et al. have shown that CFH autoantibodies isolated from early stage lung cancer patients can activate the complement system and cause cytotoxicity of tumor cells *in vitro* (Campa et al., 2015). Based on this study and the correlation of anti-CFH autoantibodies with early stage NSCLC, better outcome, and longer time to recurrence (Amornsiripanitch et al., 2010; Gottlin et al., 2022), a monoclonal anti-CFH antibody was cloned from a single peripheral B cell of a NSCLC patient. This therapeutic antibody, GT103 identifies a conformationally distinct epitope of CFH located in SCR 19 domain (Bushey et al., 2016). It exhibits growth inhibitory activity *in vivo* in several different cancer models (Bushey et al., 2016; Bushey et al., 2021; Bushey et al., 2023; Saxena et al., 2023). Further, it enhances CDC of rituximab-resistant malignant B cells (Winkler et al., 2017). GT103 has shown potential as a therapeutic agent in cancer (Clarke et al., 2022).

**FIGURE 1:**
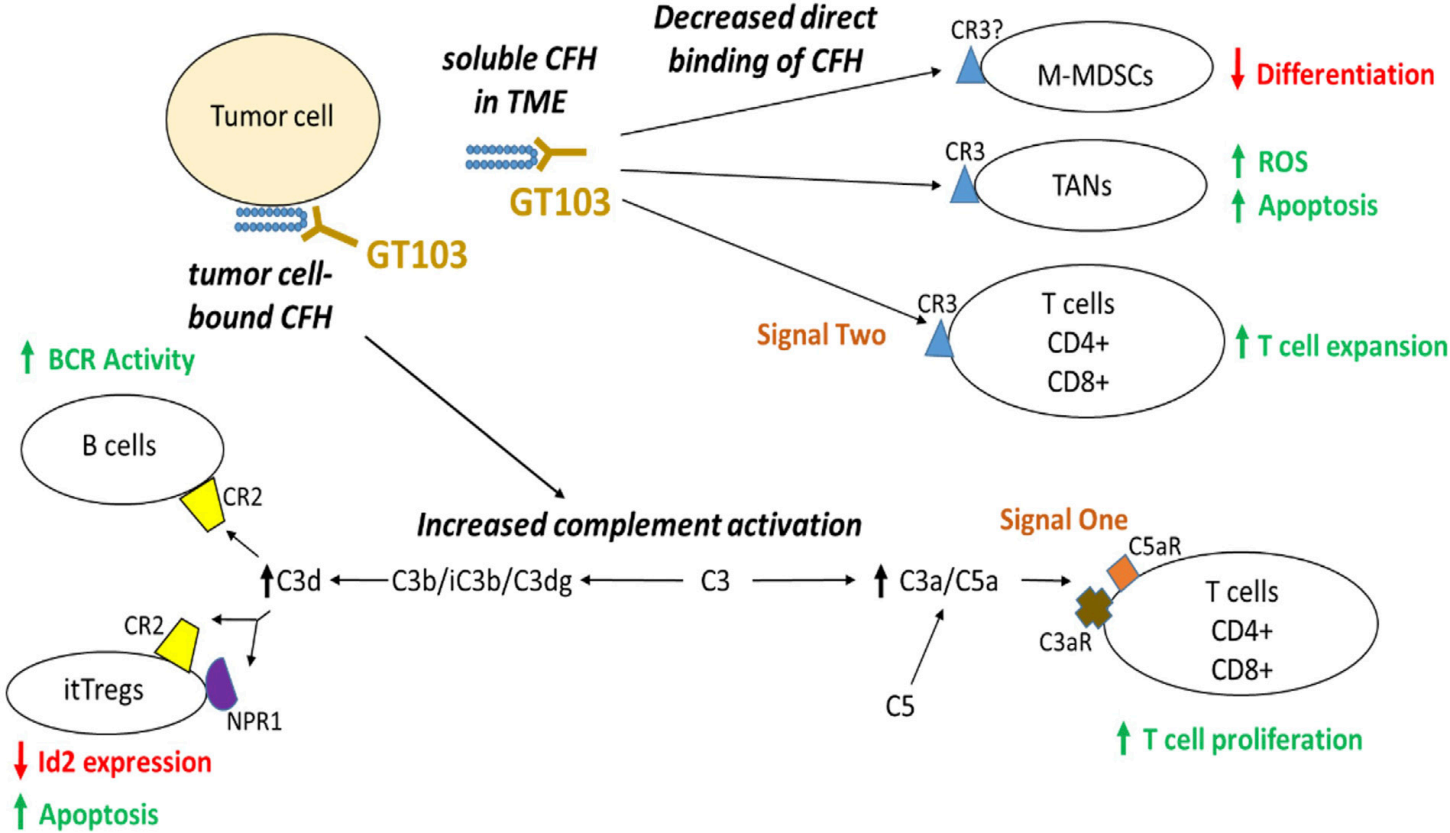
Anti-tumor mechanism of GT103. CFH in the TME inhibits antitumor immunity by two major modes of actions: direct binding to its receptor CR3 on TANs and activated T cells as well as possibly M-MDSCs, and indirectly through suppressing complement activation. GT103 blockade of CFH actions in the TME results in reduced M-MDSC differentiation, increased apoptosis of itTregs and TANs, as well as enhanced expansion of effector CD4^+^/CD8^+^ T cells and activated B cells.

Studies on the mechanism of action of GT103 show that it overcomes CFH-mediated complement evasion in the TME. In summary, GT103 modulates the TME and limits tumor growth via the following processes (summarized in Figure 1). 1. GT103 causes complement activation through the CP and induces CDC of cancer cells both *invitro* and *invivo* (Bushey et al., 2016; Bushey et al., 2021; Bushey et al., 2023). 2. GT103 enhances antigen presentation and T cell expansion. GT103 treatment increases antigen specific CD4^+^ and CD8^+^ T cells in the TME in a syngeneic model of lung cancer (Saxena et al., 2023). Further, it increases the influx of effector T and B cells along with DCs and also leads to increased activation of B cell receptor pathways, mechanisms crucial for antigen presentation (Bushey et al., 2023; Saxena et al., 2023). 3. GT103 inhibits Treg activation and expansion. GT103 treatment decreases itTregs in the TME possibly by inhibiting their activation and inducing their apoptosis (Saxena et al., 2023). 4. It creates a favorable TME by modulating immune cell trafficking and cytokine production, counteracting the effect of CFH. GT103 reduces the influx of immunosuppressive tumor associated neutrophils, MDSCs, and itTregs. It further reduces serum levels of pro-inflammatory and pro-tumorigenic cytokines (Saxena et al., 2023). 5. GT103 inhibits tumor-associated macrophage polarization to an immunosuppressive phenotype as evidenced by decreased M2-type macrophages in the TME (Saxena et al., 2023). 6. Further, it inhibits hepatocellular carcinoma (HCC) tumorigenesis and metastasis driven by tumor extracellular vesicles overexpressing CFH (Mao et al., 2020). GT103 can also target CFH-expressing tumor-derived exosomes for destruction via innate immune mechanisms via Fc interactions with C1q or, presumably Fc gamma receptors on macrophages (Bushey et al., 2021). Thus, by promoting complement activation and immune cell recruitment to the TME, the anti-CFH antibody GT103 has demonstrated efficacy in suppressing tumor growth in preclinical models. The antibody is currently being evaluated as monotherapy in a phase Ib clinical trial (Clarke et al., 2022) and in combination with Keytruda in phase II for advanced, refractory/relapsed NSCLC patients.

In addition to using monoclonal antibodies to target CFH, small molecule inhibitors that can disrupt CFH’s interaction with tumor cells and immune cells can be created as therapeutic agents and utilized as single agents or in combination with other therapies to improve their efficacy. Furthermore, CFH-based vaccines can be designed to encourage the immune system to recognize and destroy CFH-overexpressing tumor cells. CFH peptides or fusion proteins can be used as antigens in these vaccines to elicit specific immune responses against CFH-expressing tumor cells. To improve immunogenicity, CFH-based vaccines can be coupled with adjuvants or immune-stimulatory drugs. Adoptive cell therapy with CFH-specific immune cells such T cells or natural killer (NK) cells can also be utilized to target and eradicate CFH-expressing tumor cells. To improve specificity and cytotoxicity against tumor cells, these immune cells can be modified to express CFH-specific receptors such as T cell receptors (TCRs) or chimeric antigen receptors (CARs). Furthermore, gene editing techniques, such as CRISPR-Cas9, can be used to alter CFH expression in tumor cells and could potentially be used as anti-cancer therapy. These immunotherapeutic approaches have the potential to increase immune surveillance and promote anti-tumor immune responses and need to be explored as anti-CFH therapy for cancer. In addition, treatment outcomes for cancer could be greatly enhanced by combining CFH-targeted treatments with other therapeutic techniques. Multiple facets of tumor growth and immune evasion can be addressed by combinations with immunotherapies, chemotherapy, and targeted treatments. By releasing the inhibitory effects of CFH and further activating anti-tumor immune responses, immune checkpoint inhibitors, such as anti-PD-1/PD-L1 or anti-CTLA-4 antibodies, can be used in conjunction with CFH-targeted therapies to increase the effectiveness of immune checkpoint blockade (Ajona et al., 2019b; Ricklin et al., 2019; Saxena et al., 2023). CDC of rituximab resistant malignant B cells from CLL patients can be augmented by GT103 (Winkler et al., 2017) and CDC of human lung tumor cell lines by the anti-PD-L1 drug avelumab can be augmented by GT103 (Saxena et al., 2023). Further, chemotherapeutic drugs can be used in conjunction with CFH-targeted therapies to take advantage of the immunomodulatory effects of CFH inhibition while also specifically targeting tumor cells. In addition to promoting immune-mediated tumor clearance, this combined approach can synergistically increase the cytotoxic effects on tumor cells.

## 5 Future perspectives and challenges

A thorough knowledge of the molecular pathways underpinning CFH-mediated immune evasion is essential to take full advantage of the therapeutic potential of CFH and requires further investigation. Targeted therapy development will benefit from understanding the intricate interactions between CFH, complement proteins, immune cells, and the TME. While anti-CFH therapy has shown promise in preclinical and early clinical trials, there are significant obstacles to its use in cancer treatment. Targeting a major component of the immune system, such as CFH, might have unintended consequences, such as upsetting immunological balance, which can lead to autoimmune reactions and increased susceptibility to infections. The development of atypical hemolytic uremic syndrome (aHUS) is a major concern, as evidenced by a finding that shows autoantibodies against the CFH SCR 19-20 domains are correlated with aHUS (Blanc et al., 2012; Durey et al., 2016). As a result, the challenge is to develop anti-CFH agents that specifically target cancer cells while protecting healthy cells that express CFH. Furthermore, the duration and dosage of anti-CFH medication can influence the likelihood of autoimmune responses. Prolonged or high-dosage treatment may increase the risk of immune system dysregulation; thus, the dose must be carefully regulated.

Furthermore, the effectiveness of anti-CFH therapy might also depend on the type of cancer and individual patient characteristics. Some tumors may not respond as well to this sort of treatment, as the targeting epitope may be absent. Identifying the patients who would gain the most from anti-CFH therapy is still a challenge. Patient selection based on biomarkers, the patient immune status, or genetic profile is crucial, although it is still in its early stages. Furthermore, patients with pre-existing autoimmune disorders could experience progressive or new symptoms when exposed to anti-CFH therapy and may not be suitable candidates.

In addition, cancer cells may develop resistance to anti-CFH treatment over time as is seen with other targeted medicines. This has the potential to result in therapy failure and disease progression. To achieve the most favorable results, anti-CFH therapy may need to be combined with other treatment modalities such as chemotherapy, immunotherapy, or radiation therapy. The successful implementation of CFH-targeted medicines depends on selecting the right patient populations, tailoring treatment plans, and addressing potential off-target consequences.

## 6 Conclusion

The complement system is a complex but important part of innate immunity. Through its control of the complement system, interactions with immune cells, and effects on the TME, complement factor H contributes significantly to immune evasion in cancer. Developing efficient strategies to combat immunological evasion while driving an effective adaptive immune response in cancer requires an understanding of the intricate interactions between CFH and numerous immune system elements. As a therapeutic strategy to boost anti-tumor immune responses and better patient outcomes, targeting CFH shows promise. To fully investigate the therapeutic potential of CFH as a therapeutic target in cancer immunotherapy, additional study and clinical trials are required.
